# Community pharmacists and chronic pain: A qualitative study of experience, perception, and challenges

**DOI:** 10.1080/24740527.2020.1749516

**Published:** 2020-09-24

**Authors:** Hamed Tabeefar, Feng Chang, Martin Cooke, Tejal Patel

**Affiliations:** a School of Pharmacy, University of Waterloo, Kitchener, Ontario, Canada; b School of Public Health and Health Systems, University of Waterloo, Waterloo, Ontario, Canada

**Keywords:** pharmacists, chronic pain, opioid, community

## Abstract

**Background**: Patients suffering from chronic pain frequently ask pharmacists for advice.

**Aims**: This study was prompted by inadequacies in the available body of literature reporting on pharmacists’ experiences with providing care for patients with chronic pain in the community setting.

**Methods**: A qualitative investigation of Ontario community pharmacists’ experiences was carried out. Participants were interviewed using a semistructured guide. Interviews were analyzed using thematic analysis, influenced by grounded theory.

**Results**: This study revealed that pharmacists were knowledgeable and empathetic toward patient concerns. Challenges in their role included financial factors, patient access to multimodal treatment options, potential for harm associated with opioid use, inadequate monitoring, and gaps in training.

**Conclusions**: This study reports community and Family Health Team pharmacists’ experiences caring for patients with chronic pain and perceptions of their professional role, including strengths and limitations, and identifies perceived challenges in the health care system.

## RÉSUMÉ

**Contexte**: Les patients souffrant de douleur chronique demandent fréquemment conseil aux pharmaciens.

**Objectifs**: Cette étude a été motivée par les insuffisances observées dans la littérature disponible sur les expériences des pharmaciens en matière de soins aux patients souffrant de douleur chronique en milieu communautaire.

**Méthodes**: Une enquête qualitative sur les expériences des pharmaciens communautaires de l'Ontario a été réalisée. Les participants ont été interrogés à l'aide d'un guide semi-structuré. Les entretiens ont été analysés à l'aide d'une analyse thématique, influencée par la théorie ancrée.

**Résultats** : Cette étude a révélé que les pharmaciens étaient bien informés et faisaient preuve d'empathie à l'égard des préoccupations des patients. Les difficultés rencontrées dans leur rôle comprenaient des facteurs financiers, l'accès des patients à des options de traitement multimodales, les risques associés à l'utilisation d'opioïdes, un suivi inadéquat et des lacunes dans la formation.

**Conclusions** : Cette étude rapporte les expériences de pharmaciens communautaires et de l'équipe de santé familiale dans la prise en charge des patients souffrant de douleurs chroniques, les perceptions de leur rôle professionnel, y compris leurs forces et leurs limites, et recense les défis perçus dans le système de santé.

## Introduction

Although medication dispensing is central to the role of community pharmacists in Canada, pharmacists are evolving their scope of practice by increasingly taking on responsibilities such as adapting prescriptions, therapeutic monitoring, conducting medication reviews, providing patient education, and, in some jurisdictions, prescribing new treatment.^1–3^ Pharmaceutical care services can reduce negative therapeutic outcomes by 53% to 63% through decreased drug-related morbidity and mortality and improved patient adherence.^4,5^

Chronic pain, commonly defined as pain lasting longer than three months, affects approximately one in five adults.^6^ It affects all age groups, ethnicities, and genders and impacts the quality of life of individuals and their family members.^7^ In a Canadian study, nearly half of the patients with chronic pain reported having suffered from chronic pain for more than ten years, and one third reported very severe pain intensity.^8^ Additionally, chronic pain conveys a considerable economic burden to the health care system; annual related health care costs are estimated to exceed US$6 billion in Canada^9^ and US$635 billion in the United States, which surpasses the costs of treating cancer, heart disease, and diabetes.^10^

Pharmacotherapy is a key component of chronic pain management, and patients frequently seek advice from pharmacists.^8,11,12^ However, because chronic pain is often associated with several comorbidities such as depression, anxiety, and sleep disorders, treatment necessitates the use of complex drug regimens, which increases the risk of drug interactions and side effects. As highly accessible medication experts, pharmacists can assist patients with individualizing their therapy based on their comorbid conditions, concurrent medications, and other specific needs, expectations, and goals. Pharmacists routinely provide information on opioid therapy, benefits and risks, overdose identification and management, and proper storage and disposal.^13^ Patients in pharmacy settings feel ready to discuss and accept written information on related risks such as substance use disorders.^14^ Other services that pharmacists can provide include optimizing patients’ therapy through pain assessment, medication review, monitoring for interactions, ensuring appropriate dosing, and consulting on switching or tapering of medications such as opioids.^13^ However, the pharmacist’s role in the management of chronic pain remains largely unstructured and underinvestigated. Gaps in knowledge exist, with fewer than half (48%) of pharmacists reporting being familiar with the Canadian opioid guidelines and only 52% able to state the recommended opioid watchful dose.^7^

The beliefs and attitudes of both pharmacists and patients can affect the care provided and the outcomes of that care. Treatment outcomes have been shown to be affected by patients’ initial beliefs and expectation of success for a given pain treatment,^15^ and there is evidence that those beliefs can be influenced by the information and advice presented to patients about their pain by a health care provider.^16^ For example, in chronic lower back pain, both the patients’ perceptions of their condition and health care providers’ beliefs and attitudes, such as whether a patient is likely to be difficult to treat, can influence treatment decisions.^17,18^

Although pharmacists’ perceptions and beliefs regarding chronic pain might contribute to the formulation of treatment decisions and to the information provided to patients, the content of those perceptions and beliefs has not been investigated. Therefore, the aim of this study is to explore the perceptions, beliefs, and experiences of pharmacists regarding patients with chronic non-cancer pain. This includes pharmacists’ understanding of their role in providing care to patients with chronic pain, their perceptions of patients with chronic pain, and their experience of communication with prescribers in the context of providing care to patients with chronic pain.

## Methods

### Design

Due to the lack of previous research on the topic, we chose a qualitative, exploratory study design to address the question under study and used one-on-one interviews with a small sample of community pharmacists to facilitate a detailed examination of pharmacists’ perspectives.

### Participants and Settings

We used a purposive sampling strategy to recruit Ontario-licensed pharmacists who were actively practicing in a community pharmacy or on a Family Health Team. In the province of Ontario, Family Health Teams are practice settings that can include nurse practitioners, registered nurses, social workers, and dietitians as well as physicians and pharmacists. We recruited participants by means of a recruitment poster and invitation letters sent by e-mail through the Ontario College of Pharmacists’ database of pharmacists who had provided consent to be contacted for research purposes. We screened participants for eligibility, excluding those not practicing or practicing in other settings, such as hospitals, nursing homes, or pharmaceutical companies. No incentives were provided for participation in the study. We obtained approval of the study by the University of Waterloo Human Research Ethics Committee and written consent from all study participants.

### Data Collection and Analysis

We collected data through individual semistructured interviews. This format provides the interviewer with enough flexibility to explore emergent lines of inquiry, while also keeping the interview fairly focused on the general topic. A major benefit of this method is that the flexible format helps develop rapport and gain participants’ trust by being fairly conversational, as well as allowing researchers to explore participants’ responses in depth.^19^

Our approach was influenced by grounded theory, which has become a widely used framework for qualitative research in the social and health sciences.^20^ A core element of the grounded theory approach is that theoretical propositions or relationships are developed throughout the data collection and analysis process, rather than proposed a priori to be tested. Our research was also intended to be exploratory and fairly descriptive rather than concerned with developing a theoretical framework. However, it was important for us to approach the research without strong expectations about pharmacists’ experience and to let themes emerge from the research process. Because three of the authors, including the main interviewer, are pharmacists, it was impossible not to have some preconceptions about pharmacists’ perceptions. Contrary to the original formulations of grounded theory, we consulted previous research in the area in advance.

A primary interview guide was developed by research team members based on the previous body of literature in chronic pain management. In this stage, having both pharmacist and non-pharmacist team members helped avoid unintentionally incorporating assumptions about pharmacy practice into the questions. This interview guide was then revised after the first three interviews with pharmacists. The initial and final interview guides covered seven main topics for discussion; an introduction with specific questions about the pharmacists’ current practice and length of experience, their feelings towards patients with chronic pain, perceived concerns, thoughts and beliefs regarding the use of opioids for chronic pain, experience with communication with healthcare providers within the context of chronic pain treatment, challenges encountered and pharmacist training regarding chronic pain (Table 1).

**Table 1.** Semistructured interview guide.

Question category	Description
Introduction (Questions 1a–1c)	Please tell me about yourself. For example, how long have you been working as a pharmacist? What is your understanding of chronic pain? For example, how do you define it? Approximately, what percentage of your patients have chronic pain disorders (e.g., headache, low back pain, diabetic neuropathy, etc.)?
Feelings (Question 2)	Generally speaking, how do you feel when you are approached by patients with chronic pain disorders? What is different with other patients, if anything? How do you feel when asked for over-the-counter medications that do not need prescription? How do you feel when patients have a prescription for pain (including opioid medications)?
Concerns of patients about pain and opioids (Question 3)	In your experience, what are the main concerns of patients with chronic pain, pain intensity (disability), difficulty in communication with health providers, medications, and perception of other people? How do you address these concerns?
Opioids (Questions 4a–4d)	What are your concerns about dispensing opioids? For example, intoxication safety, adverse effects, dependence and addiction. How do you address these concerns with patients and prescribers? How do you respond when you are approached by an opioid-seeking patient? What is your reaction? What concerns have patients expressed about use of opioids? For example, stigma, cost, addiction, and side effects. Do you see resistance to use or overuse of opioids among your patients? How do you discuss these issues with patients? Are you aware about tools that guides these discussions?
Communication (Questions 5a–5c)	What is your experience with communicating your recommendations to prescribers? In your opinion, how effectively can a pharmacist work with other health professionals (prescribers) in improving care for patients with chronic pain? How do you communicate with prescribers or other health professionals about your patients with chronic pain? How does a prescriber communicate with you about their concerns related to their patients with chronic pain? What concerns are usually discussed with other health professionals about patients with chronic pain? For example, about prescribers’ opioid prescribing practices, safety issues (type of opioids), efficacy, legal or financial issues, and coverage. What are the barriers to communicating your concerns with prescribers? Are prescribers difficult to reach, not receptive, or uncommunicative about the treatment plan? How do you feel (what is your experience) about communication with prescribers in this concern? When do you refer patients to other health care professionals? How do you refer patients to other health care professionals?
Challenges (Questions 6a–6b)	What are the main complexities in dealing with/dispensing a prescription for patients with chronic pain? In comparison to other patients, what barriers do you encounter with patients with chronic pain? For example, fear of dispensing opioids, safety, and legal issues. In comparison to other patients, what differences do you encounter in patients with chronic pain? In your opinion, to what extent can you help patients with chronic pain improve or relieve their pain? In your opinion, what role should pharmacists play in helping patients with chronic pain?
Education (Questions 7a–7c)	In your opinion, do pharmacists need more training regarding chronic pain in order to fully address patients’ needs? What kind of training do you think you would need? For example, scientific issues (knowledge, patient assessment), communication skills, legal issues, and pharmacy practice models. Why? Do you think (What are your ideas about) the educational material in theory and practice that were/are taught in school about chronic pain can address the skills required for the practice of chronic pain management? What are your suggestions for improving or advancing pharmacists’ skills and proficiency in providing or dealing with chronic pain management? In school? In practice?

We conducted interviews from June to September 2016, either in person or by telephone, according to pharmacists’ schedules. A single interviewer conducted all but one interview; two team members were present for the first interview. Interviews lasted 40 to 90 min and were audio recorded. We transcribed the recordings verbatim using NVivo qualitative analysis software v11 for coding (QSR International Pty Ltd. Version 11, 2015).

Our analysis method was also influenced by the constant comparison method of grounded theory.^21^ As in grounded theory, each interview transcript was coded immediately after collection to identify salient themes, with the coding structure modified as new themes emerged. We followed this open coding with axial and selective coding to identify and refine new themes.^21^ However, the analysis is best described as thematic analysis because the emphasis was not on the development of theory as such, and the themes that were developed were mainly descriptive.^22^

To help ensure rigor in the data collection and analysis and to avoid unintentional bias due to our own perspectives and experiences, we made use of the diversity of the research team in several ways. The first interview was conducted by two team members, a pharmacist and a non-pharmacist social scientist, who then met to discuss the contents of the interview guide and initial impressions regarding possible themes. After being transcribed, the first two interviews were independently coded by two researchers, who then compared their coding structures to test the conceptual validity and completeness of the set of themes that had been identified. The subsequent interviews were then coded by the main interviewer after transcription. The coding of these interviews was reviewed by a third team member for consistency. The entire research team reviewed and commented on the final set of themes.

Grounded theory generally calls for theoretical sampling, in which additional data are collected based on the emerging themes in the analysis and researchers direct the sampling procedure to further develop the theory.^19^ As with much research in health care services, our purposive sampling was not strictly theoretical.^23^ Recruitment continued, concurrent with the analysis, but additional participants were not specifically selected for theoretical reasons but to ensure that thematic saturation was reached; that is, until no substantial new information about themes was generated by additional interviews.^21^

We conducted three in-person interviews and nine one-on-one telephone interviews. We reached data saturation by the ninth interview, and the final three interviews did not identify any additional themes. In previous research exploring perspectives of community pharmacists and other medical personnel, saturation has been met with similar sample sizes (<20 participants).^24,25^ In this case, the relative consistency of the views of the pharmacists and the straightforward nature of the interview questions likely contributed to saturation being reached with this small sample.

## Results

We interviewed 12 pharmacists. The mean age of participants was 46 years (range 27–63). Of the 12 participants, 66% were female, and participants had been practicing pharmacists for 19 years on average: 17 years for those with Family Health Team backgrounds (1 participant was from a Family Health Team and 2 participants had a combination of Family Health Team and community pharmacy experience) and 20 years for community pharmacists (the remainder of participants). Most participants had a bachelor of pharmacy degree (8 participants) and only 17% of participants had residency training (2 participants) or doctor of pharmacy degrees (2 participants). Topics discussed and themes identified from the qualitative analysis are shown in Table 2.

**Table 2.** Topics discussed and themes identified in qualitative interviews with Ontario pharmacists.

Item	Description
Perceptions of patients with chronic pain	Empathy Patients’ desire for opioids Patients’ desire to be understood
Perceived role of pharmacists in providing care to patients with chronic pain	Assessment and management of pain Patient education Comprehensive approach to pain management Responsibility to minimize risk of opioid misuse
Factors that are believed to hinder perceived role	Sale of medication Cost Wait times Knowledge gaps Opioid misuse
Experiences with prescribers in context of providing care to patients	Reasons for contacting prescribers Communication barriers Inadequate monitoring leading to multiple prescriptions and adverse events

Participant pharmacists did not differ greatly in their understandings of what constituted chronic pain. When asked how they would define chronic pain, almost all mentioned the core characteristics of chronic pain: unknown etiology and chronicity (exceeding 3–6 months).^26^ Some expanded their definition of chronic pain by commenting on associated issues such as anxiety, depression, disability, and impaired quality of life.

P3: Chronic is associated with long-term, so we’re dealing with pain that is due to a physical condition that’s … maybe lasting for a couple of months. We’re dealing with a full spectrum of symptoms from anxiety, from depression, from even organic effects on blood pressure and sugar control.

### Pharmacists’ Perceptions of Patients with Chronic Pain

With only one exception, pharmacists described feelings of empathy toward patients with chronic pain and expressed their thoughts on their patients’ perceived desires (e.g., to be understood, to be given pain medications, etc.). In their experience, the distress of pain and disability was visible in their patients’ appearance. This perception induced a feeling of empathy toward patients with chronic pain. The pharmacist participants thought that the most distressing part of chronic pain for patients was inadequate pain control intertwined with the chronicity of the pain.

P5: [They] look like they have a black cloud over their head. They always look sad, they look depressed. They look unhappy. The people with true chronic pain, their quality of life, their joy in life, is not sustained.

P7: They want to know what they are; they have an anxiety because having pain is not normal for them. They don’t know if it’s good or bad. So, that’s if it’s a new pain. But if it’s not a new pain and they have been having pain for a long time, then their first mention—it’s inconveniencing them.

P9: They have many concerns, you know, stemming from their pain. Not just that their pain is not controlled but that they cannot function and, you know, have a good quality of life.

Pharmacists perceived that patients seeking drugs sometimes place pressure on physicians to prescribe opioids. In these cases, patients insist on having opioids prescribed for their pain and might even threaten their prescribers.

P5: I had one doctor tell me, he had a patient who was in her 80s, and he said, “She’s going to fire me, as a doctor! She’s been my patient for over 40 years and she threatened to fire me!” So, you know, I think that doctors do get a problem with their patients and they don’t want to upset them either, you know, if you have a nice guy as a doctor.

Participants also believed that because pain is a subjective feeling, it might not be well understood by those who have not experienced chronic pain. The subjective characteristic of pain was described as being emotionally challenging for patients because they frequently encounter skepticism or misunderstanding related to their pain. Pharmacists reported that patients with chronic pain often expect their families, as well as society in general, to believe their accounts of pain and to validate their experiences and expect their health care providers to hear their concerns and to do their best to control their pain.

P1: I think there is something about being seen, being heard, being understood, being validated, and having a place where they are believed. And some days I think that that’s the therapy and that’s in and of itself.

P3: Lots of these patients are concerned with how they feel when they approach their health care provider or their pharmacist for their prescriptions.

### Perceived Role of Pharmacists in Providing Care to Patients with Chronic Pain

The pharmacists in this study described their role in providing care to patients with chronic pain as including several dimensions. These included the assessment and management of pain, especially when assisting patients with selecting over-the-counter analgesics or dispensing prescribed medications for pain. Respondents reported routinely inquiring about the severity, duration, and possible etiology of pain when assessing patients’ pain to determine the best course of action for the patient, including recommending nonprescription options for treating pain.

Pharmacists in this study also saw their role as being part of a comprehensive approach to pain management, which included collaboration with prescribers and other health care professionals. All participating pharmacists stated that if a referral were necessary they would refer the patient to his or her family physician. Study participants frequently spoke of the physical, psychosocial, vocational, and social aspects of chronic pain and believed that treatment of chronic pain should not be limited to prescribing of medications. Chronic pain and its associated health conditions, such as depression and anxiety, that impact patients’ lives should, they felt, be addressed by a variety of health care professionals, including physiotherapists, psychotherapists, nurses, social workers, and occupational therapists.

P8: I think that it’s something that needs to be addressed, perhaps holistically—more than just prescribing painkillers.

Finally, pharmacists felt that they played a significant role in advising and educating patients about pain management, including face-to-face consultations with a focus on education about pain, medications used in the treatment of pain, and nonpharmacological measures such as keeping a pain diary, lifestyle changes, and better understanding additional resources, such as websites.

Unrealistic expectations about chronic pain management and the inadequate use of alternatives for opioids were two parameters that pharmacist participants felt often led to inappropriate use of medications, particularly opioids. Because most patients do not achieve complete resolution of their pain even with opioid medications, several participants felt that patients should receive education to help them to have realistic expectations about their pain and that chronic pain management should focus on functionality.

P1: Are you moving more? Are you doing more in your day? No? Do you think this is really giving you much more in your quality of life and you being able to participate? If you’re not improving function, then [the medication is] not worth having.

### Factors Believed to Hinder Pharmacists’ Performance of Their Perceived Role

There were a number of factors that pharmacists believed may hinder their ability to fulfill their perceived role. These included financial factors, particularly the fact that the sale of medications is the primary source of income for community pharmacists. This was seen as sometimes being a barrier to pharmacists being effective in finding nonpharmacological options. For example, one described how, if they refused the sale of large quantities of opioid products that are available for purchase without a prescription, patients might take their business elsewhere.

P10: Now this patient will approach the nearby pharmacy and will buy whatever he wants, but we missed our customer.

The cost of medications was also seen as a barrier to pharmacists fulfilling their role in care of patients with chronic pain. Because non-opioid therapeutic options such as pregabalin, or non-drug interventions such as physiotherapy, are expensive and typically not covered by government health care plans, opioids are a less costly option for the management of chronic pain, encouraging physicians to prescribe opioids. Pharmacist participants also emphasized that patients with chronic pain often did not have adequate support from the government or the framework within which the health care system exists, as evidenced by the lengthy wait times for consultations with pain specialists.

Although the necessity of a comprehensive approach was clear to participants, they believed that this was best achieved in medical centers in which health care professionals work together to serve patients with chronic pain. They believed that even though chronic pain is a multidimensional health condition that requires a multimodal approach through collaboration of several health care providers, there is insufficient budget allocated by the government to cover multimodal collaborative care services.

P9: Treatment is complex and multifactorial and drugs are a small part of a successful treatment plan and the other parts are kind of expensive—things like physio, occupational therapy. And it might, it’s kind of in a way, it’s hard to measure too. So, you know, like it’s easy for the province to look at wait times for hip replacement. Although I guess they could look at wait times for chronic pain clinics, this is like they do not open a can of worms.

Another important barrier to pharmacists fulfilling their role in care for patients with chronic pain was knowledge among pharmacists themselves. Almost all pharmacist participants believed that there was a wide range in pharmacist knowledge about chronic pain management and that, in general, pharmacists needed more training in several areas, including pain assessment, pharmacologic and nonpharmacologic treatments, and communication skills. Differences in knowledge between pharmacists was thought to lead on occasion to contradictory recommendations to patients, adversely affecting pharmacists’ credibility and patients’ trust. In the perspective of some participants, this inconsistency might arise from pharmacists’ different educational experiences, and this was thought to differ with time since graduation. Several pharmacists described a difference in knowledge between pharmacists who had graduated within the previous few years and members of cohorts that graduated 10 or 20 years prior. However, participants felt that these knowledge gaps could be bridged by continuous education programs.

P4: Just in case people ask a question and go to another pharmacy down the street—which often happens—and somebody says to them something completely different. And that doesn’t look as professional.

P2: We learn it all in school, but some of us graduated 2 years ago, some 10 years ago, some 20, some 30. So, a refresher would be good to boost that confidence and to do that.

P6: If you have a very good knowledge of pain and the management, then it will definitely help you to communicate better with physicians in terms of also recommending alternatives or you have a better idea about the dosing and other alternative options as well. So, it’s a combination of both—the knowledge and the communication together.

Gaps in knowledge related to regulation were another factor perceived to hinder pharmacists in performing their role. Pharmacists expressed a need for more knowledge and training in legal issues associated with narcotics. Respondents reported that many pharmacists are not confident in their ability to react in an optimal manner to forgeries or to report misconduct of health care professionals to authorities.

P5: Pharmacists aren’t taught to recognize suspicious prescribing methods, and we aren’t taught what to do about it.

P4: Like, if someone came in, how would you catch it or how would you respond? Because no one taught us, like do I call the police, do I—like even now, to this day I don’t know if I’m legally obliged to call the police if we catch them. I would think yes, but the thing is how would you do so? Like you need to run through some scenarios, because when it actually does happen to you in real life.

Another factor perceived to hinder pharmacists’ performance of their role was the risk of opioid misuse disorder. The potential risk for misuse was the most frequently stated concern of pharmacists related to opioid use and was associated with a strong sense of responsibility to minimize risks for patients. From their perspective as gatekeepers for opioid use, the pharmacists in our study believed that they had a responsibility for the health and safety of their patients as well as society. Participants felt a need to balance risk of misuse with providing relief from pain for their patients and described themselves as exercising caution in monitoring the appropriateness of medications.

P3: We’re being asked to be the gatekeepers and making sure that we’re dispensing properly.

### Experiences with Prescribers in Context of Providing Care to Patients

From the pharmacists’ perspectives, inadequate monitoring of efficacy and safety for patients receiving opioid therapy stem from several factors: insufficient time, inadequate communication systems between health professionals, pressure from patients seeking medication, and pharmacy finances (e.g., sale of medication etc.). They believed that family physicians do not currently allocate enough time to addressing the multiple issues related to the use of opioids; our respondents felt that physicians often did not have sufficient time to review medical records of patients who were prescribed opioids to determine potential for risk of misuse. They believed that, in the context of insufficient monitoring, strategies to reduce the potential of opioid misuse should be used, such as limiting the permitted quantity of opioids in a prescription and/or limiting prescribing to health care providers with specific qualifications in opioids.

P9: I don’t think we’re very systematic in assessing for addiction. The pain specialist that I work with, she is systematic—she does that with every patient—but family doctors are not.

P3: The other one is limiting quantities; I think that’s extremely important. Physicians need to be more aware of quantities. Physicians need to be more aware of refill frequency.

Almost all pharmacists in this study stated that they had good communication with physicians, preferring to communicate with physicians by fax, with the exception of one Family Health Team pharmacist who preferred face-to-face communication. Reasons for contacting a physician included correcting errors in prescriptions, such as improperly ordering refills for opioids, incorrect amounts of medications, typos, or a lack of physician signature. Pharmacists also communicated with physicians to discuss drug interactions, to confirm opioid dose conversions, and to answer questions from physicians inquiring about the availability of medications and available dosage forms. Despite this good communication, there was a lack of a dedicated communication system enabling pharmacists and physicians to update patient information. A common example cited was patients’ use of multiple walk-in clinics to obtain multiple prescriptions, without any of the prescribers or pharmacists being aware of the overlap of service.

Participants believed that communication between pharmacists and physicians had improved over the previous 20 years. All pharmacist respondents had positive comments regarding their communication with physicians, although they did indicate that barriers for effective communication with physicians continue to exist. Almost all participant pharmacists believed that physicians were generally not very accessible, particularly those physicians working in hospitals for whom direct office contact information is often unavailable. Pharmacists perceived some physicians to be uncooperative, and they identified several characteristics they thought typified these uncooperative physicians. Pharmacists perceived younger physicians as well those who were trained in a team-focused environment as more communicative and receptive to pharmacists’ comments. Additionally, pharmacists felt that the personality of the individual physician played a role, explaining that some people are less communicative by nature. Pharmacists generally thought that physicians who were empathetic and spent more time on their patients were also more likely to be receptive to pharmacists’ comments. As one commented,

P5: If they are an empathetic doctor who gives each patient more than their 5 or 10 minutes, then usually they’re willing to accept suggestions. But if they’re a “you get 5 minutes and you’re out the door/one-problem doctor,” then I usually find that they are not responsive to any suggestions from a pharmacist.

Trust between pharmacists and physicians was seen as playing a pivotal role in communication. Pharmacists believed that trust develops over time and that it was the responsibility of pharmacists to make physicians familiar with the profession of pharmacy and their scope of practice, as well as to provide physicians with appropriate recommendations.

P1: So, pharmacists are often bound by little picky details that they have to ensure that are done, and physicians may not be aware of that. And with a doctor who is in a high-stress area—and pharmacists are too—they won’t realize why they are getting these messages and why these things need to be changed.

## Discussion

To the best of our knowledge, this is the first qualitative study to investigate pharmacists’ perceptions and experiences with providing care to patients with chronic pain in a community setting. Participating pharmacists were knowledgeable about chronic pain and the challenges of providing care to patients with chronic pain. Adequate monitoring, appropriate use of medications and drug information resources, and patient education were identified as factors important for the effective and safe management of chronic pain.

Patients’ unrealistic treatment expectations, inadequate access to alternative treatments for opioids, and the lack of a comprehensive approach to pain management were identified as factors that can impede management. Because there is a fine line between effective pain control and impaired functionality due to opioid safety risks, unjustified long-term opioid therapy can be associated with poor self-rated health, being unemployed, higher use of the health care system, and reduced quality of life.^27^ In contrast, key opioid treatment goals include pain relief, improved quality of life, and improved functional capacity.^27^ Patients who have function-focused expectations about chronic pain management report improved experience of pain and reduced risk of side effects and addiction.^28–30^ This suggests that educating patients about the characteristics of chronic pain and its management and defining pain relief along with functionality as the ultimate treatment goals will lead to realistic expectations about treatment and result in a more balanced use of medications and potentially reduced dependence on opioids.

Participating pharmacists also recognized and were empathetic to patients’ concerns about lack of pain control, as well as lack of family, social, and government supports, such as inadequate access to expert health care and collaborative team-based care (e.g., pain clinics), which add to the distress of patients with chronic pain. These beliefs were well founded and are supported by the results of a four-year follow-up study that found that two thirds of patients do not receive adequate pain management^31^ and an eight-year follow-up study that reported that only 34.6% of patients were pain free.^32^

Driven by the ongoing expansion of pharmacists’ scope of practice and the development of pharmaceutical care, it is expected that the role of pharmacists will continue to move from medication-oriented dispensing toward a more comprehensive patient-centered model of practice in which pharmacists use their expertise to assist patients in addressing their pain, rather than simply dispensing medications.^33,34^ There is evidence that this expanded role is effective in providing better outcomes for patients. Pharmacists can help reduce the strength of pain medication and frequency of use while maintaining pain management.^35^ Delivering patient education can reduce pain intensity and reduce adverse events,^36^ decrease the cost of pain management,^37^ and help reduce the frequency of harmful medication combinations (e.g., opioids combined with benzodiazepine).^38^

Pharmacists are still at the early stages of this transition. In this study, pharmacists felt that their ability to perform these expanded roles was limited by various barriers. One important barrier was the risk of potential harm associated with opioid use and confidence in managing this risk. Canada is the world’s second largest per capita consumer of opioids.^39^ Despite the lack of data to support long-term efficacy and safety of opioids for chronic pain,^28^ the pharmacists in this study were very concerned that the use of opioids for chronic non-cancer pain is continuing to increase. Similar findings have been found in a 2011 survey of Ontario pharmacists, who voiced a concern for patients on opioids.^13^ Previously surveyed Canadian pharmacists have also expressed low confidence in their ability to identify patients with potential prescription drug misuse and addiction.^40^ Targeted curriculum education and training postgraduation were suggested as potential solutions to help build pharmacist capacity in this area.

Recent Canadian guidelines for opioids for chronic non-cancer pain and draft regulations to expand the scope of pharmacists’ practice encourage pharmacists to take more responsibility for appropriate use of medications and patient monitoring for common diseases and potentially risky medications like opioids.^33,34^ Adequate monitoring ensures that signs of aberrant drug behavior will be recognized as soon as possible by pharmacists, who can then initiate early intervention strategies.^41^ However, pharmacists consider their workload in community pharmacies, gaps in communications with prescribers, and the fact that medication sales are the primary source of income for pharmacies as barriers to managing potential misuse and diversion of opioids at the pharmacy level. One suggested solution is reimbursement for the documenting and monitoring of opioid prescriptions, to empower pharmacists to better assume the shared role of stewards for opioid medications.

Although a multimodal approach is recommended by best practice, participants felt that lack of access is a barrier to formulating a comprehensive approach. This is reflected in previous research showing that lack of insurance coverage for alternative medications or treatment approaches is a core concern that can lead to inappropriate pain management.^42,43^ A number of non-opioid medications and interventions have demonstrated effectiveness in chronic pain disorders.^44,45^ Moreover, non-opioid medications and interventions for the management of chronic pain can produce better functional outcomes than opioids, being outperformed only by strong opioids for pain relief.^44,46–48^ Because chronic pain is disabling, with up to 25% of patients losing their jobs,^46^ expanded access with financial support is vital to widening therapeutic options for pain management.

### Study Limitations

This study captured the perspectives and opinions of Ontario pharmacists working in a community setting or in a Family Health Team. These opinions may not be representative of pharmacists in other provinces because the pharmacist role varies provincially. Additionally, the use of two different interview modes (in person and telephone) might have influenced the results, although research suggests that such an effect might not be substantial.^49^

## Conclusion

To conclude, this study describes community and Family Health Team pharmacists’ experiences caring for patients with chronic pain and perceptions of their professional role and identifies perceived challenges in the fulfillment of this role. Participants suggested future action in building pharmacist capacity, making system-based changes to empower pharmacists in providing collaborative team-based care, and improving funding for nonmedicinal and non-opioid pain treatments.

## Data Availability

Data are available prior to ethics board–mandated deletion. Please send request to Hamed Tabeefar: tabeefar@gmail.com
